# Membrane Binding Promotes Annexin A2 Oligomerization

**DOI:** 10.3390/cells9051169

**Published:** 2020-05-08

**Authors:** Anna Lívia Linard Matos, Sergej Kudruk, Johanna Moratz, Milena Heflik, David Grill, Bart Jan Ravoo, Volker Gerke

**Affiliations:** 1 Institute of Medical Biochemistry-Center for Molecular Biology of Inflammation, University of Münster, Von-Esmarch-Str. 56, D-48149 Münster, Germany; linardma@uni-muenster.de (A.L.L.M.); sergej.kudruk@uni-muenster.de (S.K.); milena.heflik@web.de (M.H.); david.grill@uni-muenster.de (D.G.); 2 Organic Chemistry Institute, University of Münster, Corrensstrasse 40, D-48149 Münster, Germany; j_mora01@uni-muenster.de (J.M.); b.j.ravoo@uni-muenster.de (B.J.R.)

**Keywords:** annexin A2, microdomain, cross-linker, quartz crystal microbalance with dissipation monitoring (QCM-D)

## Abstract

Annexin A2 (AnxA2) is a cytosolic Ca^2+^ regulated membrane binding protein that can induce lipid domain formation and plays a role in exocytosis and endocytosis. To better understand the mode of annexin-membrane interaction, we analyzed membrane-bound AnxA2 assemblies by employing a novel 3-armed chemical crosslinker and specific AnxA2 mutant proteins. Our data show that AnxA2 forms crosslinkable oligomers upon binding to membranes containing negatively charged phospholipids. AnxA2 mutants with amino acid substitutions in residues predicted to be involved in lateral protein–protein interaction show compromised oligomer formation, albeit still being capable of binding to negatively charged membranes in the presence of Ca^2+^. These results suggest that lateral protein–protein interactions are involved in the formation of AnxA2 clusters on a biological membrane.

## 1. Introduction

Biological membranes can segregate into microdomains of defined lipid and protein composition that serve diverse but yet very specific tasks. One category of these microdomains, often referred to as lipid rafts, is enriched in cholesterol as well as sphingomyelin species present in the extracellular leaflet and in certain cases phosphatidylinositol (4,5)-bisphosphate [PI(4,5)P_2_] present in the cytoplasmic leaflet. Rafts are particularly well studied in the plasma membrane of eukaryotic cells, where they serve as assembly and transmission platforms in outside-in as well as inside-out signaling and also regulate membrane trafficking events to and from the plasma membrane (for review see [1,2,3]). To function in these processes, rafts have to be highly dynamic, both with respect to lipid and protein composition as well as size, thus they assemble into larger structures and also disassemble again on a rapid time scale. Dynamic assembly/disassembly is driven by intrinsic properties of the raft lipids and proteins and is affected, among other things, by cholesterol content and degree of fatty acid saturation in the incorporated phospho- and sphingolipids. Importantly, raft dynamics are also controlled by membrane-associated proteins that function by binding to raft lipids or proteins and affect their properties and distribution. These associated proteins are often regulated in their membrane association and thus raft-controlling properties enable cells to rapidly respond to certain stimuli by altered membrane microdomain assembly. One important regulatory event is a change in intracellular Ca^2+^ concentration and a number of raft-associated proteins whose membrane interaction is regulated by Ca^2+^ (for review see [4]). Annexins are a family of such Ca^2+^ regulated proteins that bind to acidic phospholipids in the cytoplasmic leaflets of cellular membranes in a peripheral and reversible manner (for review see [5,6,7]).

Annexin A2 (AnxA2) is a member of the annexin family that has been shown to associate with raft-like microdomains in certain cells and certain physiological scenarios (for review see [8]). As other annexins, it directly binds to headgroups of negatively charged phospholipids and requires the presence of these lipids in membranes (and raft domains) for high-affinity association. One such lipid is PI(4,5)P_2,_ and AnxA2 has been shown to interact with this phosphoinositide in a specific manner [9,10]. In addition to binding to PI(4,5)P_2_ and other acidic phospholipids, in particular phosphatidylserine (PS), in a Ca^2+^ regulated manner, the protein can also form two-dimensional assemblies on PI(4,5)P_2_ or PS containing model membranes and can cluster these lipids into domains [11,12,13,14]. Most likely, this lipid segregating property of AnxA2 is responsible for a function of the protein in regulating membrane-cytoskeleton contacts, cell polarity, and exocytotic granule docking and fusion [15,16,17,18,19]. However, the molecular basis underlying the phospholipid segregating properties of AnxA2, in particular the role of potential protein-protein interactions in this process, is not known.

Structurally, AnxA2 has a fold similar to all other annexins. It comprises a conserved core domain that is built of four repeats, each with five α helices, and a unique N-terminal domain mediating interactions with protein ligands. The core forms a slightly bent structure, where type-II Ca^2+^ binding sites as well as the membrane binding site are located on the convex side. A high resolution crystal structure of AnxA2 has been obtained revealing anti-parallel dimers of AnxA2 in the crystallized unit [20] and amino acid side chains residing at this dimer interface could provide lateral contacts in two-dimensional AnxA2 assemblies.

Here we have investigated the oligomeric state of membrane-bound AnxA2 to assess whether protein–protein interactions could represent the molecular mechanism underlying AnxA2-driven PI(4,5)P_2_ and PS segregation. Therefore, we developed a novel chemical crosslinker that revealed the existence of AnxA2 oligomers. These oligomers only form in the presence of Ca^2+^ and require membrane binding, thus could represent the molecular structures driving a segregation of AnxA2-bound phospholipids. Moreover, mutating residues present at the dimer interface identified positions that reside in close proximity in membrane-bound AnxA2 oligomers and could participate in oligomer formation.

## 2. Materials and Methods

### 2.1. Lipids, Chemicals

Cholesterol (Chol), 1,2-dioleoyl-sn-glycero-3-phosphocholine (DOPC), 1-palmitoyl-2-oleoyl-sn-glycero-3-phoshocholine (POPC), 1-palmitoyl-2-oleoylsn-glycero-3-phosho-L-serine (sodium salt) (POPS), and 1,2-dioleoyl-sn-glycero-3 [phosphoinositol-4,5-bisphosphate](triammonium salt) (PI(4,5)P_2_) were purchased from Avanti Polar Lipids Inc. (Alabaster, AL, USA). Lipids and cholesterol were dissolved in chloroform/methanol (1:1, *v*/*v*), except for PI(4,5)P_2_ that was dissolved in chloroform/methanol/water (20:9:1 *v*/*v*). Other chemicals were purchased from Applichem (Darmstadt, Germany), Merck KGaA (Darmstadt, Germany), Carl Roth GmbH (Karlsruhe, Germany) and Sigma-Aldrich (Munich, Germany). Water was purified and deionized with a cartridge system from Millipore (18.2 MΩ). For all the SDS-PAGE and Western blots the marker PageRuler Plus Prestained Protein Ladder from Thermo Scientific was used (Waltham, MA, USA).

### 2.2. Crosslinker Synthesis

**Figure 1:**
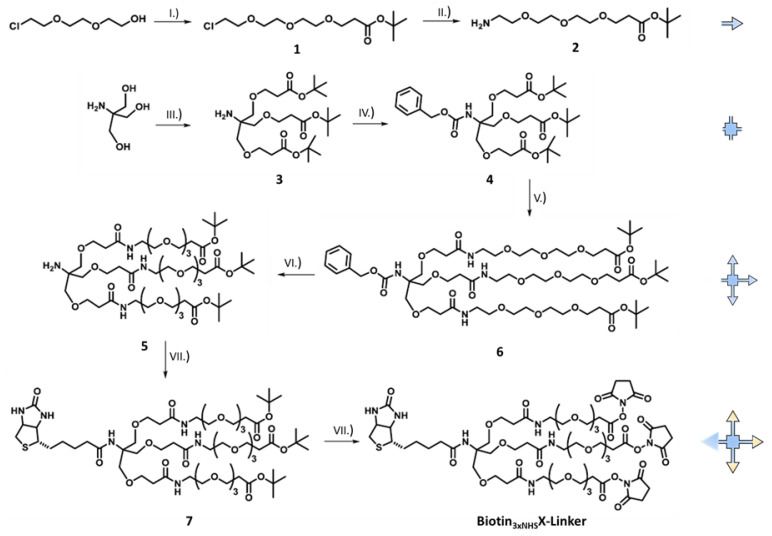
Synthesis of the Biotin_3xNHS_X-Linker: (**I.**) *t*-butylacrylate, sodium, THF, 16h, 13%, (**II.**) 1. NaN_3_, DMF, 70 °C, 2d, 2. PPh_3_, H_2_O, THF, 4d, quant., (**III.**) *t*-butylacrylate, NaOH, DMSO, 15 °C and then warm up to rt, 20h, 23%, (**IV.**) benzyl chloroformate, Na_2_CO_3_ (aq.), DCM, 4d, 94%, (**V.**) 1. TFA, DCM, 2h, 2. (**2)**, DIPEA, PyBoP, DMF, 24 h, 69%, (**VI.**) H_2_, Pd/C, MeOH, 3d, 88%, (**VII.**) D(+)Biotin, DIPEA, PyBoP, DMF, 18h, 38%, (**VII.**) 1. TFA, DCM, Toluol, 2h, 2. *N*-hydroxysuccinimide, DCC, THF, 3d, quant. See SI and Figures S1 and S2 for details.

Biotinyl *N*-Tris((2-(2.5-dioxopyrrolidin-1-yl propionate triethyleneglycolamido) ethoxy) methyl) methylamide, herein referred to as Biotin_3xNHS_X-Linker, was synthesized as described in detail in the Supplementary Information (SI). Briefly, spacer synthesis started with 2- (2- (2-chloroethoxy) ethoxy) ethanol, which was converted into the ester (**1**) in a MICHAEL reaction with *t*-butyl acrylate and sodium in tetrahydrofuran (THF) (Figure 1). The chloride (**1**) was then mixed with sodium azide (NaN_3_) and the azide obtained was reduced to the amine-terminated spacer with triphenylphosphine. In the convergent procedure, Tris (hydroxymethyl) aminomethane (THAM) was converted with *t*-butyl acrylate and sodium hydroxide (NaOH) in THF to the triple-functionalized amine (**3**), which was then protected with benzyl chloroformate (**4**). This protective group shows stable behavior in the case of tri-fluoro acetic acid (TFA) initiated acidic hydrolysis. This was followed by selective TFA deprotection of the ester-protected hydroxyl group with subsequent peptide coupling of the molecule (**4**) and the spacer (**2**). In the next step, the *N*-Cbz protective group was removed under reductive conditions using hydrogen and palladium on activated carbon (Pd/C). The amine (**5**) obtained was further processed under peptide coupling conditions with D(+)biotin, diisopropylethylamine (DIPEA), and benzotriazol-1-yl-oxytripyrrolidinophosphonium hexafluorophosphate (PyBOP) to yield the biotin-functionalized product (**7**). The activated crosslinker (Biotin_3xNHS_X-linker) was obtained after deprotection of the ester units and direct functionalization of the carboxylic acids with N-hydroxysuccinimide (NHS) and N, N′-dicyclohexylcarbodiimide (DCC) in THF.

### 2.3. Liposome Preparation and Co-Sedimentation

Lipids dissolved in chloroform/methanol were mixed at the desired molar ratio and composition. Chloroform was evaporated under a stream of nitrogen and traces of solvent were removed in vacuum for 4 h. Lipid films were stored at 4 °C until use. Liposomes were formed by hydration of the lipid film in a PBS −/− buffer. Small unilamellar vesicles (SUVs, 50 nm) or large unilamellar vesicles (LUVs, 100 or 200 nm) were obtained by extrusion through polycarbonate membranes (Avanti Polar Lipids Inc.). SUVs were employed in quartz crystal microbalance with dissipation monitoring (QCM-D) experiments to facilitate vesicle rupture that occurs following vesicle coalescence on the sensor surface and results in the formation of a stable bilayer [21]. LUVs were used in liposome co-sedimentation and crosslinking experiments to ensure efficient co-pelleting and prevent rupture more often observed with high curvature vesicles. 

Co-sedimentation experiments employed liposomes composed of POPC:Chol:POPS (60:20:20) with a defined size of 200 nm at a final concentration of 1 mg/mL. Liposomes were incubated for 1h at 4 °C with the desired AnxA2 derivative at a liposome/protein ratio of 10/1 (µL/µg) in PBS −/− buffer containing 1 mM CaCl_2_. Ultracentrifugation (UC) was performed to pellet the liposomes (96600 *g*, 4 °C for 20 min), the supernatant was collected and the pellet resuspended in 500 µL of PBS with 1 mM CaCl_2_, followed by 20 min incubation at 4 °C. After a second UC, the supernatant was collected and the pellet was resuspended in 500 µL of PBS with 5 mM EGTA and incubated for 30 min at 4 °C. A third UC yielded a supernatant (EGTA eluate) and a pellet that was resuspended in 500 µL of PBS with 5 mM EGTA. All fractions were analyzed via SDS-PAGE and immunoblotting with AnxA2-specific antibodies [22].

### 2.4. QCM-D Measurements

Quartz Crystal Microbalance with Dissipation (QCM-D) analysis was performed as described before [21,23] using a Q-Sense E4 QCM-D (Q-Sense, Gothenburg, Sweden) equipped with four temperature controlled flow cells in a parallel configuration connected to a peristaltic pump (Ismatec IPC, Glattbrugg, Switzerland), at a flow rate of 80.4 µL/min. A bilayer was established by fusion of SUVs composed of POPC/DOPC/Chol/POPS/PI(4,5)P_2_ (37:20:20:20:3). Binding measurements were performed at 20 °C in HBS buffer supplemented with 250 µM Ca^2+^ and AnxA2 constructs at 50 nM. 250 µM Ca^2+^ and a relatively complex lipid mixture were chosen in the QCM-D experiments to directly compare the results to our previous data obtained by QCM-D analysis of AnxA2 and other AnxA2 mutants [21,24]. Frequency and dissipation shifts of the 7th overtone resonance frequency of the quartz sensor (QSX 303, 50 nm SiO2, 4.95 MHz) were recorded. OriginPro v. 9.1 (OriginLab Corp.) was used for data analysis. 

### 2.5. Crosslinking of AnxA2

LUVs (100 nm) composed of POPC:DOPC:Chol:POPS (40:20:20:20) were used at 3.33 µg/µL in HBS pH 7,4. Control #1 contained only the AnxA2 derivative (60 µg) in 1 mM CaCl_2_, i.e., a reaction in the absence of membranes, while control #2 contained a mixture of AnxA2 (60 µg) with 100 µg of LUVs, 5 mM EGTA, and 0.3 mM Biotin_3xNHS_X-Linker, i.e., a reaction in the absence of Ca^2+^. The actual Ca^2+^/crosslink sample consisted of LUVs (100 µg), AnxA2 (60 µg), 1 mM CaCl_2_, and 0.3 mM Biotin_3xNHS_X-Linker. A Ca^2+^ concentration of 1 mM was used in these experiments to ensure efficient phospholipid binding of the protein. All components were mixed with exception of the Biotin_3xNHS_X-Linker, which was added after 30 min, and incubation was then continued for another 30 min while shaking. The reaction was stopped with 5× PAGE sample buffer without β-mercaptoethanol. For a better separation in the gel, 3 µL of a 100 mM EGTA solution was added to each sample. Samples were kept for 15 min at RT before analysis by 10% SDS-PAGE. Gels were stained with Coomassie Brilliant Blue. Quantification of crosslinked oligomer bands was achieved by gating the area in the stained gel lane above the position of AnxA2 dimers in all samples and relating its intensity to that of the monomer band in the respective sample. In this quantification, the actual dimer band was excluded because AnxA2 species migrating at the dimer position in SDS-PAGE, which do not reflect the physical state of the protein in solution and most likely form during SDS sample preparation, have been observed before [22]. They would mask an association not caused by the crosslink. Image Studio Lite (LI-COR Corporate Offices, NE, USA) and Graphpad Prism 4 (GraphPad Software, San Diego, CA, USA) were used for quantification.

### 2.6. Mutagenesis

The human AnxA2 cDNA carrying a substitution at amino acid 66 (glutamate-for-alanine) to establish a monoclonal antibody epitope was cloned into the pSE420 expression vector as described [11] to yield pSE420-AnxA2A66E.

AnxA2 6x (pSE420-AnxA2A66E_6x) was generated by mutating 6 amino acids in the template pSE420-AnxA2A66E using site-directed mutagenesis as described [23]. Mutations were introduced at amino acid positions: 81 (K to A), 189 (E to K), 196 (R to S), 206 (K to A), 212 (K to S) and 219 (E to K). The following primers were employed in the mutagenesis reactions: K81A_For 5′-CCAGAGAAGGACCAAAGCGGAACTTGCATCAGCAC-3′ and K81A_Rev 5′-GTGCTGATGCAAGTTCCGCTTTGGTCCTTCTCTGG-3′. E189K_For 5′-GGCTCTGTCATTGATTATAAACTGATTGACCAAGATGCTC-3′ E189K_Rev 5′-GAGCATCTTGGTCAATCAGTTTATAATCAATGACAGAGCC-3′, K206A_For 5′-CGCTGGAGTGAAGAGGGCAGGAACTGATGTTCCC-3′ K206A_Rev 5′-GGGAACATCAGTTCCTGCCCTCTTCACTCCAGCG-3′, R196S_For 5′-CTGATTGACCAAGATGCTAGTGATCTCTATGACGCTGGAG-3′ R196S_Rev 5′-CTCCAGCGTCATAGAGATCACTAGCATCTTGGTCAATCAG-3′, K212S_For 5′-CAGGAACTGATGTTCCCTCGTGGATCAGCATCATG-3′ K212S_Rev 5′-CATGATGCTGATCCACGAGGGAACATCAGTTCCTG-3′, E219K_For 5′-ATCAGCATCATGACCAAGCGGAGCGTGCCC-3′ E219K_Rev 5′-GGGCACGCTCCGCTTGGTCATGATGCTGAT-3′, (Biomers, Ulm, Germany):

AnxA2 10x (pSE420-AnxA2A66E_10x) was generated using pSE420-AnxA2A66E_6x as template by introducing 4 additional amino acid substitutions at amino acid positions: 36 (R to S), 53 (V to A), 54 (T to A) and 328 (K to A). The following primers were used: R36A_For 5′-CCTATACTAACTTTGATGCTGAGAGCGATGCTTTGAACATTG-3′ R36A_Rev 5′-GTTTCAATGTTCAAAGCATCGCTCTCAGCATCAAAG-3′, V53A_T54A_For 5′-CAAAGGTGTGGATGAGGCCGCCATTGTCAACATTTTG-3′ V53A_T54A_Rev 5′-CAAAATGTTGACAATGGCGGCCTCATCCACACCTTTG-3′, K328A_For: 5′-TAAGGGCGACTACCAGGCAGCGCTGCTGTACCTG-3′ K328A_Rev 5′-AGGTACAGCAGCGCTGCCTGGTAGTCGCCCTTAG-3′ (Biomers, Ulm, Germany). 

### 2.7. Protein Expression and Purification

For protein expression, E. coli cells transformed with the respective pSE420 plasmid were grown at 37 °C in LB medium supplemented with ampicillin to an optical density of 0.6 at 600 nm (OD600). Protein expression was then induced by addition of isopropyl β-D-1-thiogalactopyranoside to a final concentration of 1 mM. After expression for 4 h, cells were harvested by centrifugation at 5000× *g* for 10 min at 4 °C. Protein purification was performed by diethylamioethyl- and carboxymethyl-cellulose ion exchange chromatography and proteins were alkylated specifically at Cys-8 by 2-iodoacetamide treatment to prevent disulfide mediated protein crosslink as previously described [23]. 

## 3. Results

### 3.1. Protein-Protein Interaction in Membrane-Bound AnxA2

AnxA2 has been shown by atomic force microscopy to form two-dimensional assemblies on model membranes containing negatively charged phospholipids [13]. To analyze whether these assemblies are characterized by homotypic protein–protein interactions, we performed chemical crosslinking studies of membrane-bound versus soluble AnxA2. Therefore, we first developed a novel crosslinker, herein referred to as Biotin_3xNHS_X-linker, that due to its trifunctional nature should efficiently link proximal amino groups in proteins (Figure 1). Biotin_3xNHS_X-linker also contained a biotin group enabling streptavidin-mediated detection and enrichment.

**Figure 2:**
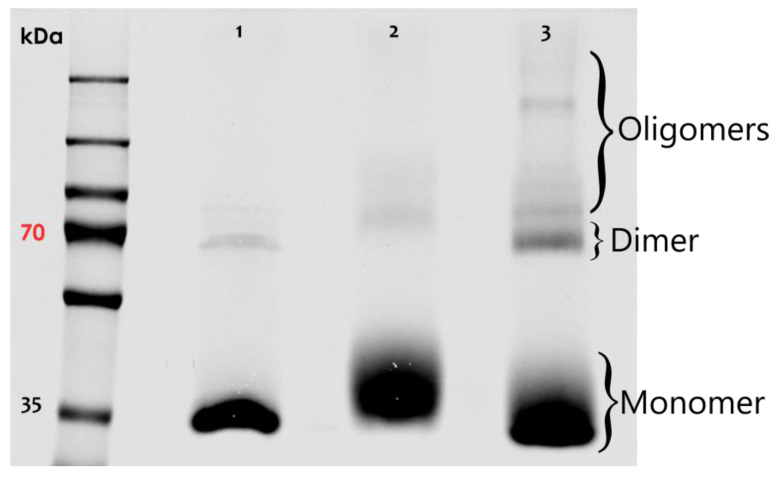
SDS-PAGE of crosslinking reactions involving alkylated AnxA2 wild-type (WT). Lane 1: Control #1 (AnxA2 WT + Ca^2+^); lane 2: Control #2 (AnxA2 WT + LUVs + EGTA + Biotin3xNHSX-Linker); lane 3: Ca^2+^/membrane sample (AnxA2 WT + LUVs + Ca^2+^ + Biotin3xNHSX-Linker). Brackets on the right indicate the positions of AnxA2 monomers, dimers, and oligomers. Dimer formation most likely occurs during sample preparation, whereas the oligomers likely present AnxA2 assemblies that form following membrane interaction and are then stabilized by the crosslinker. A representative result of *n* = 5 independently performed experiments is shown.

Biotin_3xNHS_X-linker was then used to study the nature of AnxA2 assemblies on model membranes. Purified AnxA2 (Figure S3) was treated with Biotin_3xNHS_X-linker either in the absence of membranes or following Ca^2+^-dependent binding to liposomes containing the negatively charged AnxA2-binding lipid phosphatidylserine (PS). Figure 2 shows the results of these crosslinking experiments. While a very small amount of higher molecular mass species was observed in the control reactions, i.e., AnxA2 samples in the absence of membranes or Ca^2+^, significant crosslink products indicative of oligomeric AnxA2 assemblies were generated when AnxA2 bound to PS-containing liposomes was subjected to the crosslinking reaction. Thus, our crosslink approach involving the Biotin3xNHSX-Linker indicates that membrane binding triggers the formation of AnxA2 oligomers, in which the proteins engage in lateral contacts spatially close enough to allow an effective covalent linkage by the trifunctional crosslinker.

### 3.2. Annexin A2 Oligomers on Model Membranes are Stabilized by Lateral Protein-Protein Interactions

**Figure 3:**
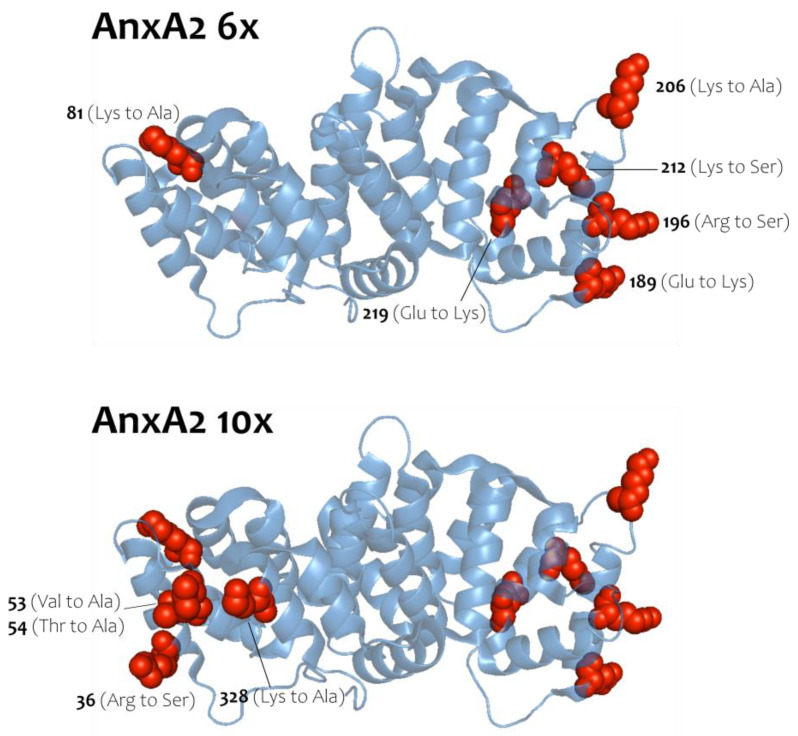
AnxA2 crystal structure highlighting mutations introduced in the AnxA2 6x and 10x constructs. AnxA2 6x top [81 (Lys to Ala), 189 (Glu to Lys), 196 (Arg to Ser), 206 (Lys to Ala), 212 (Lys to Ser) and 219 (Glu to Lys)] and AnxA2 10x bottom [36 (Arg to Ser), 53 (Val to Ala), 54 (Thr to Ala) and 328 (Lys to Ala)]. Illustrations were created using the AnxA2 crystal structure (PDB code: 1XJL).

To address the nature of the homotypic AnxA2 interaction, which occurs following membrane binding and can be stabilized by Biotin_3xNHS_X-linker, we generated two AnxA2 derivatives, in which residues predicted to participate in lateral protein-protein interactions in the crystal structure of an anti-parallel AnxA2 dimer [20] (see also pdb entry of the crystal structure of this AnxA2 dimer at 1XJL) were mutated to side chains of opposite charge or to alanine or serine (Figure 3). Importantly, the residues mutated are not part of the known type-II or type-III Ca^2+^-binding sites of AnxA2 [25] and so far have not been identified as sites of posttranslational modification. Moreover, the residues selected are characterized by polar or charged side chains and thus could engage in salt bridges and/or other ionic interactions that would favor oligomer formation. Provided that the two-dimensional AnxA2 assemblies on membranes involve these residues located on the lateral surface of the folded AnxA2 molecule, the mutants, herein named AnxA2 6x and AnxA2 10x, should show a compromised oligomer formation and thus Biotin_3xNHS_X-linker mediated crosslink. Moreover, as the mutations do not involve residues of the Ca^2+^/membrane binding sites, AnxA2 6x and AnxA2 10x are expected to retain the capability to bind to membranes containing acidic phospholipids.

**Figure 4:**
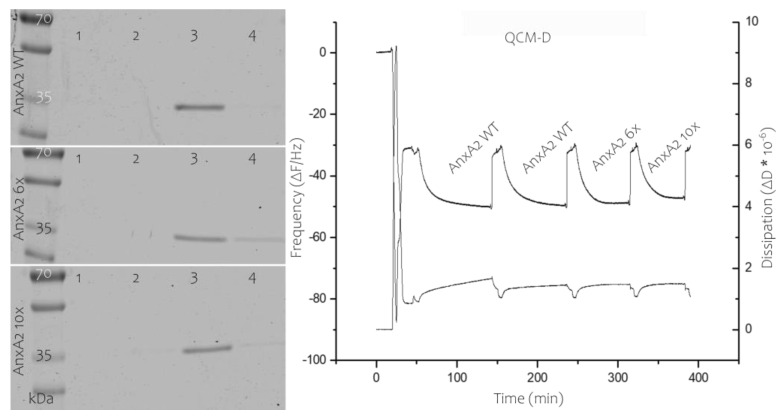
Membrane binding of AnxA2 constructs. Left, Liposome co-pelleting assay analyzed by SDS-PAGE of the different fractions. AnxA2 wild-type (WT), AnxA2 6x, or AnxA2 10x were mixed with PS-containing liposomes in the presence of 1 mM Ca^2+^. Liposomes were pelleted and the supernatant, i.e., non-bound material, was collected (lane 1). Liposomes were then washed in Ca^2+^-containing buffer, yielding a second supernatant (Ca^2+^ wash, lane 2). Subsequently, the pelleted liposomes were washed with EGTA-containing buffer resulting in release of the Ca^2+^-dependently bound material (EGTA eluate, lane 3). The final liposome pellet containing non-released protein is shown in lane 4. The gel shows a representative result of *n* = 5 independently performed experiments. Right, QCM-D measurements, frequency (as deviation from resonance frequency, ΔF) is shown in the upper recordings and dissipation (ΔD) in the lower. Following formation of a solid-supported bilayer (at a ΔF of around −30 Hz in these settings), AnxA2 WT was added in the presence of Ca^2+^, resulting in a drop in resonance frequency to approximately −49 Hz. Addition of EGTA removed all bound protein with resonance frequency returning to its initial bilayer value (−30 Hz). Recording was continued with subsequent additions (in Ca^2+^ containing buffer) and release (in EGTA containing buffer) of AnxA2 WT (to show reversibility of the reaction), AnxA2 6x, and AnxA2 10x. The QCM-D recordings were performed at least three times each for the different, independently purified AnxA2 derivatives.

AnxA2 6x and 10x were purified following the protocol developed for the wild-type protein (Figure S1). Importantly, this also involved alkylation of the exposed cysteine-8 as disulfide bridge formation involving this cysteine residue is observed under oxidative conditions [23]. The mutants were first characterized with respect to their ability to bind to membranes containing acidic phospholipids in a Ca^2+^-dependent manner by employing liposome co-pelleting and solid-supported membrane-binding assays. Figure 4 shows that AnxA2 6x and 10x effectively bind to PS-containing liposomes in the presence of Ca^2+^. As observed for the wild-type protein, this binding is fully reversible, i.e., the bound protein is released when the liposome-protein mixtures are treated with the Ca^2+^ chelator EGTA. Analysis of protein binding to solid supported membrane bilayers was carried out using a quartz crystal microbalance with dissipation (QCM-D). QCM-D is a well-established tool to evaluate and quantify protein–lipid interactions [21]. By applying SUVs, a bilayer can be formed on a sensor chip connected to a quartz microbalance and the ability of proteins to interact with this lipid bilayer can be measured via decrease in the resonance frequency of the quartz crystal that occurs as a result of mass adsorption. Importantly, due to the unique Ca^2+^-dependent and fully reversible interaction of AnxA2 with membranes, AnxA2 bound to the solid-supported membrane can be readily released from the bilayer by Ca^2+^ chelation with EGTA [24]. QCM-D recordings performed with the different AnxA2 derivatives revealed that AnxA2 wild-type (WT) and the AnxA2 6x and 10x mutants show similar binding kinetics and resonance frequency shifts (ΔΔF) of 19 Hz for AnxA2 WT, 18.5 Hz for AnxA2 6x, and 16.7 Hz for AnxA2 10x (Figure 4). Moreover, in each case the binding is fully reversible upon addition of EGTA and the dissipation increase is rather minimal indicating that a relatively rigid protein layer is formed on the solid-supported bilayer.

**Figure 5:**
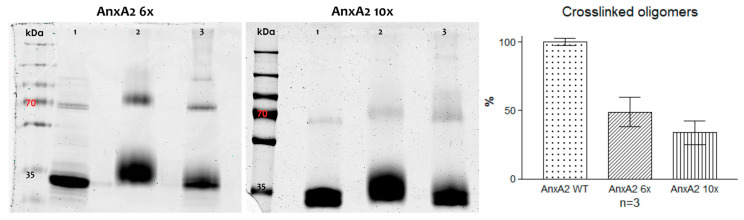
Chemical crosslinking of alkylated AnxA2 mutant proteins. Left, SDS-PAGE of crosslinking reactions involving AnxA2 6x and 10x. Each AnxA2 derivative was subjected to chemical crosslinking in the presence or absence of Ca^2+^ and LUVs. Lanes 1: Controls #1 (AnxA2 + Ca^2+^); lanes 2: Controls #2 (AnxA2 + LUVs + EGTA + Biotin_3xNHS_X-Linker); lanes 3: Ca^2+^/membrane sample (AnxA2 + LUVs + Ca^2+^ + Biotin_3xNHS_X-Linker). Right, quantification of crosslinked oligomer bands for each AnxA2 derivative (calculated in relation to the respective monomer band, see Materials and Methods). Given is the relative percentage of these oligomer bands compared to those obtained for the wild-type protein analyzed in a parallel reaction. Three independent crosslinking reactions were analyzed for each protein species and the standard error of means is indicated.

Next, the lateral side chain mutants, AnxA2 6x and AnxA2 10x, were characterized with respect to their ability to form crosslinkable oligomers following membrane binding. Mutant proteins were bound to PS-containing liposomes and proteins residing in close proximity were covalently linked employing the Biotin_3xNHS_X-linker. Figure 5 shows that the capability of forming crosslinked high molecular mass products was significantly compromised in both mutants when compared to the wild-type protein (Figure 2). A quantification of the oligomeric products revealed that higher molecular mass products representing crosslinked AnxA2 oligomers are reduced to approximately 48% and 33% for AnxA2 6x and AnxA2 10x, respectively, when compared to the wild-type protein. Thus, side chains identified in AnxA2 crystals as potential protein–protein contact sites appear to reside in close proximity in membrane-bound AnxA2, suggesting that lateral protein–protein interactions accompany the membrane association of AnxA2

## 4. Discussion

In addition to binding to membranes containing acidic phospholipids in the presence of elevated Ca^2+^ concentrations, at least some annexins can also form two-dimensional clusters or assemblies on model membranes. Such assemblies have been extensively studied in the case of AnxA5, which forms highly ordered 2D crystals on the membrane [26,27,28]. In the case of AnxA5, the building blocks in these crystalline arrays are trimers, and residues mediating and stabilizing trimer formation have been mapped [29]. In contrast to AnxA5, AnxA2 does not form such ordered crystalline arrays on model membranes, but rather appears to associate into more irregular two-dimensional assemblies of amorphous shapes [13]. Interestingly, these assemblies have been shown to segregate the AnxA2-binding phospholipids [PS, PI(4,5)P_2_] into domains underneath the bound proteins, and this activity has been suggested to underlie the function of AnxA2 in Ca^2+^-regulated exocytosis [11,12,14,16,30]. Thus, understanding the molecular basis of the AnxA2-mediated lipid segregation is of high relevance for understanding the cellular properties of the protein.

Here, we have used a chemical cross-linking approach employing a novel trifunctional compound and combined this with the characterization of AnxA2 mutant derivatives to address the nature of the 2D assemblies formed by this annexin on model membranes. Our analysis revealed that these assemblies are characterized by close protein proximity most likely involving protein–protein interactions that can be stabilized by chemical crosslinking. Moreover, we identified amino acid residues residing on the lateral surface of the folded protein that are either directly involved in the crosslink or mediate interactions between bound AnxA2 moieties that are lost in the 6x and 10x mutants. Thus, it appears likely that the assemblies containing AnxA2 and its interacting lipids [PS, PI(4,5)P_2_] are at least partly stabilized by lateral protein–protein interactions.

Our analysis benefitted from the introduction of a novel trifunctional crosslinker. Typically, chemical crosslinkers are capable of connecting spatially proximate amino acids of proteins. When selecting the crosslinker, the chemical selectivity and activity of the functional groups towards the amino acids to be linked should be taken into account. In addition, the "length/size" of the crosslinker is crucial, since it defines the maximum spatial distance of the linked amino acids. The Biotin_3xNHS_X-linker synthesized here is a homo-trifunctionalized NHS ester crosslinker, whose N-hydroxy-succinimide group can react with primary amines or alcohol groups, thus yielding high crosslink efficiency that allowed the detection of AnxA2 protein proximity in the membrane-bound form.

Although AnxA2 has been shown to cluster the lipids bound by the protein, the composition of the membrane patch underneath bound AnxA2 is not known. Most likely it also contains cholesterol as it has been shown that membrane cholesterol renders the AnxA2 binding cooperative, suggesting a cooperative nature of the assembly formation. As the protein assemblies formed by AnxA2 on the membrane are accompanied by close protein apposition, it appears plausible that the cooperation is at least in part mediated by a conformational change in the membrane-bound AnxA2, which renders it capable of undergoing protein–protein interactions. Future experiments involving streptavidin-mediated enrichment of membrane patches bound to Biotin_3xNHS_X-linker-crosslinked AnxA2 should shed some light on the lipid composition in the associated membrane. The Ca^2+^-regulated and reversible manner underlying the cluster formation of AnxA2 on cholesterol and PS/PI(4,5)P_2_ containing membranes is most likely highly relevant for its cellular function as it would support the dynamic formation of lipid platforms that could function as sites for exocytosis, and are also involved in epithelial cell polarization shown to depend on AnxA2 [15].

In summary, we introduce a novel water-soluble trifunctional NHS crosslinker, which was suitable to covalently link AnxA2 protein moieties bound to model membranes containing negatively charged phospholipids. Moreover, we could identify amino acid side chains residing on the lateral surface of folded AnxA2 that are required for efficient crosslink, and thus likely support lateral protein–protein interactions of membrane-bound AnxA2, possibly by providing salt bridges and/or other ionic interactions.

## Supplementary Materials

The following are available online at https://www.mdpi.com/2073-4409/9/5/1169/s1. Supplementary Methods including detailed protocol of the chemical synthesis of Biotin_3xNHS_X-Linker. Figure S1: ^1^H-NMR and ^13^C-NMR spectrum of Biotin_3xNHS_X-Linker, 400 MHz, CDCl_3_. Figure S2: HSQC spectrum of Biotin_3xNHS_X-Linker. Figure S3: SDS-PAGE and Western Blot of AnxA2 WT purification steps.
