# Supplementary material for: Membrane Binding Promotes Annexin A2 Oligomerization
Source: Cells. 2020 May 8;9(5):1169. doi: 10.3390/cells9051169 (PMC7291239; doi:10.3390/cells9051169)
Supplement: Supplementary file 1 [file cells-09-01169-s001.pdf]

Supplementary Information

Membrane binding promotes annexin A2 oligomerization

Anna Lívia Linard Matos[1#], Sergej Kudruk[1#], Johanna Moratz[2], Milena Heflik[1], David Grill[1], Bart Jan Ravoo[2] and Volker Gerke[1,*]

[1] Institute of Medical Biochemistry - Center for Molecular Biology of Inflammation, University of Münster, Von-Esmarch-Str. 56, D-48149, Münster, Germany;

[#] Equal contribution

[2] Organic Chemistry Institute, University of Münster, Corrensstrasse 40, D-48149 Münster, Germany;

* Correspondence: gerke@uni-muenster.de; Tel.: +49-(0)251-835-2118; Fax: +49-(0)251-835-6748

Supplementary Methods

Mass Spectrometry

Electrospray ionization (ESI) spectra were recorded either with a MicroTof (Bremen, Germany, Bruker Daltonics) or a LTQ Orbitrap XL (Bremen, Germany, Thermofisher Scientific) instrument. High molecular mass molecules were detected using MALDI (matrix assisted laser desorption ionization) carried out with an Autoflex Speed instrument (Bruker Daltonics, Bremen) equipped with a SmartBeamTM NdYAG-laser at a wavelength of 355 nm and reflector mode.

NMR Spectroscopy

Nuclear Magnetic resonance (NMR) spectra were recorded on one of the following instruments: Bruker AV 300 (USA, Massachusetts, Billerica, Bruker corporation), Bruker AV 400 (USA, Massachussets, Bruker Corporation), Valarian VNMRS 500 (Varian Inc., Paolo Alto, California, USA) or Agilent DD2 600 (USA, California, Santa Clara, Agilent Technologies) at 298 K using deuterated solvents. Chemical shift (δ) are reported in parts per million (ppm), coupling constant (*J*) are given in Hertz (Hz). Residual solvent protons were used for referencing. All spectra were analysed using MestReNova v 8.0.0 software. Signals were annotated as follows: s = singlet, d = doublet, t = triplet, q = quartet, p = pentet, m = multiplet, b = broad.

Column Chromatography and Thin Layer Chromatography

Preparative silica gel column chromatography was carried out with silica gel (mesh 40-65 µM, Merck KGaA, Darmstadt, Germany). Solvents were used without further purification and silica gel coated aluminium sheets with fluorescence indicator (Germany, Darmstadt, 60 F254 Merck KGaA) were used for thin layer chromatography. Spots were visualized by UV light at 354 or by staining with basic potassium permanganate solution, ninhydrin or EtOH/H_2SO_4.

Lyophilization

Freeze drying was carried out with an Alpha 1-2 LD plus freeze dryer (Martin Christ GmbH, Osterode, Germany). Compounds were dissolved in *dd* H2O and frozen under rotation in liquid nitrogen.

Ultra-pure water (*dd* H_2O)

Ultra-pure water with an electric resistance greater than 18 MΩ was generated with a PureLab UHQ water purification system from ELGA (High Wycombe, UK).

Ultrasonication

Ultrasonication was performed with a Sonorex RK 510 Transistor (BANDELIN electronic GmbH & Co. KG, Berlin, Germany) at a working frequency of 35 kHz at 25 °C.

Chemical Synthesis

When the experimental protocol required inert gas atmosphere, standard Schlenk techniques were used under argon atmosphere. In these case solvents were dried according to standard methods, namely: DCM over calcium hydride, THF over sodium, acetone with molecular sieves 3 Å. Other chemicals were used as purchased without further purification. Suppliers are listed below:

-Acros Organics (Thermofisher Scientific Inc. Waltham, Massachusetts, USA)

-Aldrich (Sigma-Aldrich, USA, St. Louis, Missouri, USA)

-Alfa Aesar (Alfa Aesar, Ward Hill, Massachusetts, USA)

-Carbolution (Carbolution Chemicals GmbH, Saarbrücken, Germany)

-Fluka (Sigma-Aldrich Corp. St. Louis, Missouri, USA)

-Iris Biotech GmbH (Iros Biotech GmbH, Marktredwitz, Germany)

-Merck (Merck KGaA, Darmstadt, Germany)

-Wacker (Wacker Chemie AG, München, Germany)

Tert-Butyl 3-(2-(2-(2-chloroethoxy)ethoxy)ethoxy)propanoate (**1**)

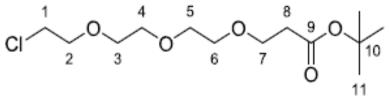

Under argon atmosphere sodium (60 mg, 2.6 mmol, 019 eq) was added to a solution of 2-(2-chloroethoxy)ethoxy)ethanol (23.2 g, 138 mmol) in THF (100 mL) and dissolved completely. Tert-butyl acrylate (22.1g, 172 mmol, 1.25 eq.) was added and the solution was stirred for 16 h. The pH was adjusted to 7 with HCl (1M, 5 mL), the solution was concentrated and brine (150 mL) was added. The mixture was extracted with EtOAc (3x100 mL), washed with brine (150 mL) and the organic phase was dried over $MgSO_4$. After removing the solvent, the product was purified by column chromatography (CH:EtOAc, 3:1 -> 2:1).

Molecular Formula: $C_{13}H_{25}ClO_5$.

Yield: 5.50 g (18.5 mmol, 13%).

Rf (EtOAc): 0.77.

^{1}H-NMR (300 MHz, $CDCl_3$): δ/ppm = 3.69 – 3.62 (m, 4H, H-1, H-2), 3.61- 3.53 (m, 10H, H-3 – H–7), 2.43 (t, J = 6.5 Hz, 2H, H-8), 1.38 (s, 9H, H-11).

^{13}C NMR (75 MHz, $CDCl_3$): δ/ppm: 170.83 (C-9), 80.43 (C-10), 71.31/70.62/70.55/70.53/70.33 (C-2 – C-6), 66.85 (C-7), 42.66 (C-1), 36.23 (C-8), 28.05 (C-11)

ESI-MS$^+$ (MeOH): $[M+Na]^+$ calc.: *m/z* = 319.1283; exp.: *m/z* = 319.1282.

Tert-butyl 3-(2-(2-(2-aminoethoxy)ethoxy)ethoxy)propionate (**2**)

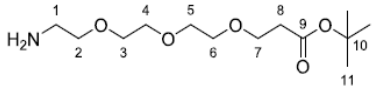

The chloride (**1**) (5.40 g, 18.2 mmol) and sodium azide (1.30 g, 20.0 mmol, 1.1 eq.) were dissolved in DMF (26.5 mL) and heated to 70 °C for 2 d. The suspension was poured into THF (27 mL) and stirred for 30 min. The reaction mixture was filtered over activated carbon and washed with THF (80 mL), The solution was purged with argon for 90 min, triphenylphosphine (5.26 g, 20.0 mmol, 1.1 eq.) was added in two portions and the reaction mixture was stirred for 3 d. Water (0.8 mL) was added and the mixture stirred for another 24 h. The organic solvent was removed under reduced pressure and the residue was taken up in water (40 mL). The precipitate was filtered off, washed with water (80 mL) and dried in high vacuum.

Molecular Formula: $C_{13}H_{27}NO_5$.

Yield: 5.05 g (18.2 mmol, quant).

^{1}H-NMR (300 MHz, $CDCl_3$): δ/ppm = 3.62 (t, J = 6.5 Hz, 2H, H-7), 3.59 – 3.50 (m, 8H, H-3 – H-6), 3.42 (t, J = 5.2 Hz, 2H, H-2), 2.77 (t, J = 5.2 Hz, 2H, H-1), 2.42 (t, J = 6.5 Hz, 2H, H-8), 1.36 (s, 9H, H-11).

^{13}C NMR (75 MHz, $CDCl_3$): δ/ppm: 170.83 (C-9), 80.44 (C-10), 73.23/70.46/70.39/70.27/70.17 (C-2 – C-6), 66.79 (C-7), 41.61 (C-1), 36.15 (C-8), 28.00 (C-11).

ESI-MS$^+$ (MeOH): $[M+Na]^+$ calc.: *m/z* = 278.1962; exp.: *m/z* = 278.1964.

Tris((2-(tert-butoxycarbonyl)ethoxy)methyl)methylamine (**3**)

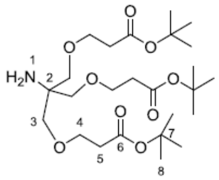

Tris(hydroxymethyl)aminomethane (4.84 g, 40 mmol) was dissolved in DMSO (8 mL) under argon atmosphere and cooled to 15 °C. Aqueous NaOH (5M, 0.80 mL) was added, followed by dropwise addition of tert-butyl acrylate (20.0 mL, 136 mmol, 3.4 eq.). The reaction mixture was stirred for 20 h and purified by column chromatography (EtOAc:CH, 2:1, 0.1% NH_4OH).

Molecular Formula: $C_{25}H_{47}NO_9$.

Yield: 4.67 g (9.25 mmol, 23%).

Rf (EtOAc:CH, 2:1, 0.1% NH4OH): 0.30.

^{1}H-NMR (300 MHz, $CDCl_3$): δ/ppm = 3.61 (t, J = 6.4 Hz, 6H, H-4), 3.28 (s, 6H, H-3), 2.41 (t, J = 6.4 Hz, 6H, H-5), 1.73 (bs, 2H, H-1), 1.41 (s, 27H, H-8).

^{13}C NMR (75 MHz, $CDCl_3$): δ/ppm: 170.86 (C-6), 80.35 (C-7), 72.77 (C-3), 67.08 (C-4), 55.94 (C-2), 36.27 (C-5), 28.06 (C-8).

ESI-MS$^+$ (MeOH): $[M+Na]^+$ calc.: *m/z* = 506.3324; exp.: *m/z* = 506.3305.

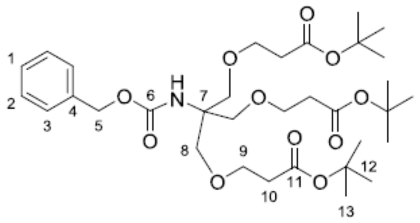

Benzyl N-tris((2-(tert-butoxycarbonyl)ethoxy)methyl)methyl-carbamate (**4**)

The amine (**3**) (4.50 g, 8.91 mmol) was dissolved in DCM (70 mL) and aqueous Na_2CO_3 (25%, 35 mL) was added under stirring. After addition of benzyl chloroformate (24 mL, 169 mmol, 19 eq.) the solution was stirred at room temperature for 4 d. The product was extracted with DCM, dried with $MgSO_4$ and the solvent was removed under reduced pressure. Column chromatography (CH:EtOAc, 4:1 -> 3:1) yielded a colorless oil.

Molecular Formula: $C_{33}H_{53}NO_{11}$.

Yield: 5.36 g (8.39 mmol, 94%).

Rf (CH:EtOAc, 2:1): 0.54.

^{1}H-NMR (300 MHz, $CDCl_3$): δ/ppm = 7.37 – 7.16 (m, 5H, H-1, H-2, H-3), 4.96 (s, 2H, H-5), 3.70 – 3.46 (m, 12 H, H-8, H-9), 2.36 (t, J = 6.4 Hz, 6H, H-10), 1.36 (s, 27H, H-13).

^{13}C NMR (75 MHz, $CDCl_3$): δ/ppm: 170.92 (C-11), 155.19 (C-6), 136.82 (C-4), 128.48/128.06 (C-2, C-3),

127.97 (C-1), 80.55 (C-8), 69.46 (C-12), 67.17 (C-9), 66.20 (C-7), 58.81 (C-5), 36.31 (C-10), 28.18 (C-13).

ESI-MS$^+$ (MeOH): $[M+Na]^+$ calc.: *m/z* = 662.3511; exp.: *m/z* = 662.3506.

Benzyl N-tris((2-(tert-butyl propanoate triehyleneglycol amido)ethoxy)methyl) methyl-carbamate (**5**)

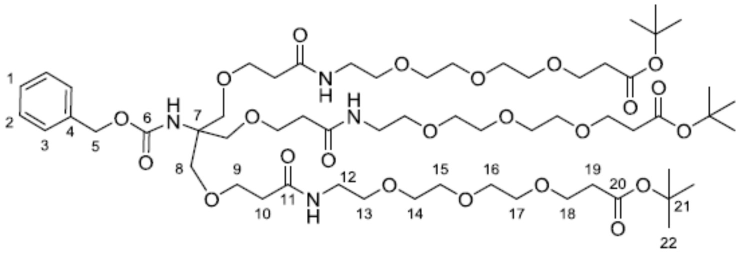

Under argon atmosphere (**4**) (1.072 g, 1.67 mmol) was stirred in DCM:TFA (1:1, 20 mL) for 1h and dried under reduced pressure. The procedure was repeated, TFA was removed in high vacuum and the residue was dissolved in DMF (peptide grade, 20 mL). DIPEA (15 mL, 87 mmol, 60 eq.), PYBOP (4.54 g, 8.72 mmol, 6 eq) and the amine (**2**) (2.25 g, 8.11 mmol, 5.6 eq) were added and the reaction was stirred at room temperature for 24 h. Silica column chromatography (EtOAc:MeOH, 7:3) yielded a yellow viscous oil.

Molecular Formula: $C_{60}H_{104}N_4O_{23}$.

Yield: 1.44 g (1.15 mmol, 69%).

Rf (CH:EtOAc, 2:1): 0.65.

^{1}H-NMR (300 MHz, $CDCl_3$): δ/ppm = 7.36 (d, J = 3.9 Hz, 5H, H-1, H-2, H-3), 5.05 (s, 2H, H-5), 3.77 – 3.50 (m, 48H, H-8, H-9, H-13 – H-18), 3.45 – 3.11 (m, 9H, H-10, H-12), 2.62 – 2.48 (m, 6H, H-19), 2.45 (t, J = 6.0 Hz, 3 H, H-10), 1.46 (s, 27H, H-22).

^{13}C NMR (75 MHz, $CDCl_3$): δ/ppm: 174.39 (C-20), 173.47 (C-11), 138.20 (C-4), 133.13/133.00 (C-2, C-3), 130.15 (C-1), 82.22/71.21/71.14/70.98 (C-13 – C-18), 70.55)C-9), 68.70 (C-5), 67.82 (C-8), 67.73 (C-7), 40.36 (C-12), 37.53/37.00 (C-10, C-19), 28.42 (C-22).

ESI-MS$^+$ (MeOH): $[M+2Na]^{2+}$ calc.: m/z = 647.3438; exp.: m/z = 647.3431.

Tris((2-(tert-butyl propanoate triethylenglycol amido)ethoxy)methyl) methylamine (**6**)

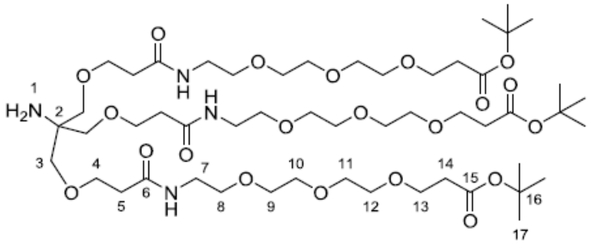

Compound (**5**) (1.44 g, 1.15 mmol) was dissolved in MeOH (20 mL) and purged with argon for 45 min. Palladium on activated carbon (10 wt%, 145 mg, 136 µmol, 0.12 eq.) was added and the mixture was stirred for 3 d under H_2 atmosphere. The suspension was filtered through Celite and washed with MeOH. The product was obtained after solvent removal.

Molecular Formula: $C_{52}H_{98}N_4O_{21}$.

Yield: 1.13 g (1.01 mmol, 88%).

Rf (EtOAc:MeOH, 7:3): 0.08.

^{1}H-NMR (300 MHz, $CDCl_3$): δ/ppm = 5.05 (s, 2H, H-1), 3.66 (dt, J = 17.4, 4.9 Hz, 48H, H-3, H-4, H-8 – H-13), 3.44 – 3.26 (m, 6H, H-7), 2.49 (dt, 21.1, 6.0 Hz, 12H, H-5, H-14), 1.46 (s, 27H, H-17).

^{13}C NMR (75 MHz, $CDCl_3$): δ/ppm: 174.34 (C-15), 173.14 (C-6), 81.96 (C-3), 71.22/71.14/71.08/71.01/70.52/68.85/67.72 (C-4, C-8 – C-13), 40.28 (C-14), 36.99 (C-7), 36.64 (C-5), 28.26 (C-17).

ESI-MS$^+$ (MeOH): $[M+H]^+$ calc.: m/z = 1115.6796; exp.: m/z = 1115.6792.

Biotinyl N-Tris((2-(tert-butyl propanoate triethyleneglycol-amido)ethoxy)methyl)methylamide (**7**)

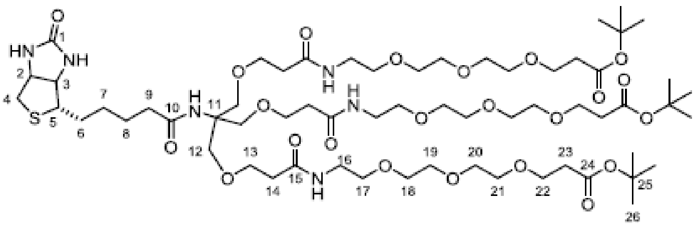

Under argon atmosphere the amine (**6**) (1.13 g, 1.01 mmol), D(+)-biotin (450 mg, 1.84 mmol, 1.82 eq.), DIPEA (4.0 mL, 23 mmol, 22.8 eq.) and PyBOP (1.20 g, 2.30 mmol, 2.28 eq.) were dissolved in DMF (peptide grade, 12 mL) and stirred at room temperature for 18 h. After removal of the solvent the product was purified by size exclusion chromatography (Sephadex LH 20, $CHCl_3$).

Molecular Formula: $C_{62}H_{122}N_6O_{23}S$.

Yield: 505 mg (0.377 mmol, 38%).

Rf (EtOAc:MeOH, 7:3): 0.65.

^{1}H-NMR (400 MHz, MeOH-d_4): δ/ppm = 4.55 – 4.47 (m, 1H, H-2), 4.33 (dd, J = 7.7, 4.4 Hz, 1H, H-3), 3.77 – 3.50 (m, 48H, H-12, H-13, H-17 – H-22), 3.43 – 3.29 (m, 9H, H-14, H-16), 3.26 – 3.18 (m, 1H, H-5), 3.16 (dq, J = 6.5, 3.7 Hz, 2H, H-4), 2.95 (dd, J = 12.7, 4.8 Hz, 1H, NH_{Biotin}), 2.72 (d, J = 12.7 Hz, 1H, NH_{Biotin}), 2.54 – 2.39(m, 9H, H-14', H-23), 2.23 (t, J = 7.1 Hz, 2H, H-8), 1.90 – 1.82 (m, 2H, H-9), 1.75 (tt, J =13.9, 7.5 Hz, 1H, H-7), 1.62 (tq, J = 14.0, 8.2, 7.5 Hz, 3H, H-6, H-7'), 1.47 (s, 27H, H-26).

^{13}C NMR (101 MHz, $CDCl_3$): δ/ppm: 176.02 (C-10), 173.89 (C-24), 172.68 (C-15), 81.67 (C-12), 71.56/71.47/71.34/71.26/70.58/70.06/68.63 (C-13, C-17 – C-22), 67.87 (C-4), 63.30 (C-5), 61.55/61.40 (C-2, C-3), 57.01 (C-9), 49.85 (C-6), 47.31 (C-8), 41.08 (C-7), 40.40 (C-23), 37.44 (C-16), 37.21 (C-14), 28.40 (C-26).

MALDI-MS$^+$ (CH_3CN): $[M+Na]^+$ calc.: *m/z* = 1363.7392; exp.: *m/z* = 1363.7391.

Biotinyl N-Tris((2-(2.5-dioxopyrrolidin-1-yl propionate triethyleneglycolamido)ethoxy)methyl)methylamide (**Biotin$_{3xNHS}$X-Linker**)

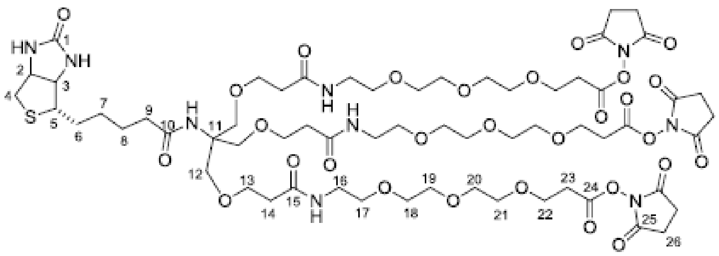

Under argon atmosphere (**7**) (500 mg, 370 µmol) was stirred in DCM/TFA (1:1, 2 mL) for 1 h and dried under reduced pressure. The procedure was repeated twice, TFA was removed in high vacuum and the residue was dissolved in THF (20 mL). DCC (762 mg, 3.7 mmol, 10eq.) and N-hydroxysuccinimide (425 mg, 3.7 mmol, 10 eq.) were added and the reaction was stirred at room temperature for 3 d. The urea byproduct was filtered off and washed with THF. The filtrate was concentrated and purified by size exclusion chromatography (Sephadex LH 20, $CHCl_3$).

Molecular Formula: $C_{62}H_{97}N_9O_{29}S$.

Yield: 540 mg (0.369 mmol, quant).

^{1}H-NMR (400 MHz, MeOH-d$_4$): δ/ppm = 6.85 (t, J = 5.4 Hz, 3H, N-H), 6.76 (t, J = 5.7 Hz, 1H, N-H), 4.44 (dd, J = 7.8, 4.5 Hz, 1H, H-3), 4.26 (ddd, J = 9.6, 7.8, 4.5 Hz, 1H, H-2), 3.77 (t, J = 6.3 Hz, 6H, H-22), 3.71 – 3.52 (m, 30H, H-12, 18-21), 3.65 – 3.61 (m, 6H, H-13), 3.49 (t, J = 5.4 Hz, 6H, H-17), 3.35 (q, J = 5.4 Hz, 6H, H-16), 3.09 (q, J = 6.7 Hz, 1H, H-5), 2.83 (t, J = 7.3 Hz, 2H, H-9), 1.69 – 1.50 (m, 4H, H-6, H-8), 1.44 – 1.32 (m, 1H, H-7).

^{13}C NMR (101 MHz, CDCl$_3$): δ/ppm: 173.53 (C-10), 171.44 (C-15), 169.18 (C-25), 166.76 (C-24), 163.69 (C-1), 70.09, 70.28, 70.43, 70.61 (C-18 - C-21), 69.73, 69.19 (C-12), 67.36 (C-13), 65.64 (C-22), 61.79 (C-2), 60.11 (C-3), 59.66 (C-11), 55.62 (C-5), 40.45 (C-4), 39.17 (C-16), 36.40 (C-14), 35.91 (C-9), 32.08 (C-23), 27.90 (C-6), 27.77 (C-7), 25.56 (C-26), 25.33 (C-8).

IR (neat): ν·cm^{-1} = 648 (m), 810 (w), 1068 (s), 1084 (s), 1204 (s), 1366 (m), 1427 (w), 1543 (m), 1651 (m), 1732 (s), 1781 (w), 2870 (m), 2924 (w), 3302 (br).

MALDI-MS$^+$ (CH_3CN): $[M+Na]^+$ calc.: *m/z* = 1464.6186; exp.: *m/z* = 1464.6156.

Supplementary Figures

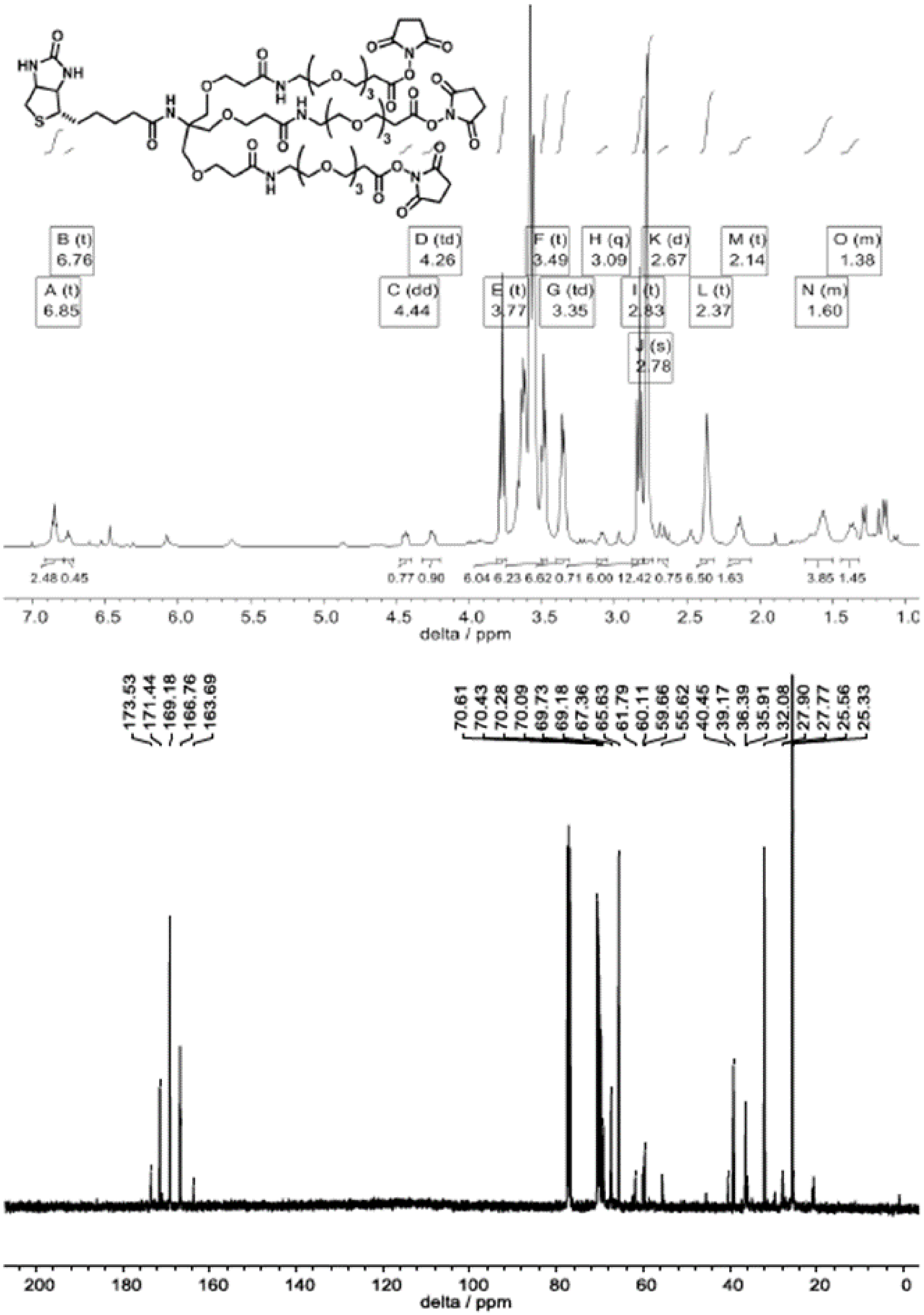

Figure S1. ^{1}H-NMR and ^{13}C-NMR spectrum of Biotin$_{3xNHS}$X-Linker, 400 MHz, $CDCl_3$.

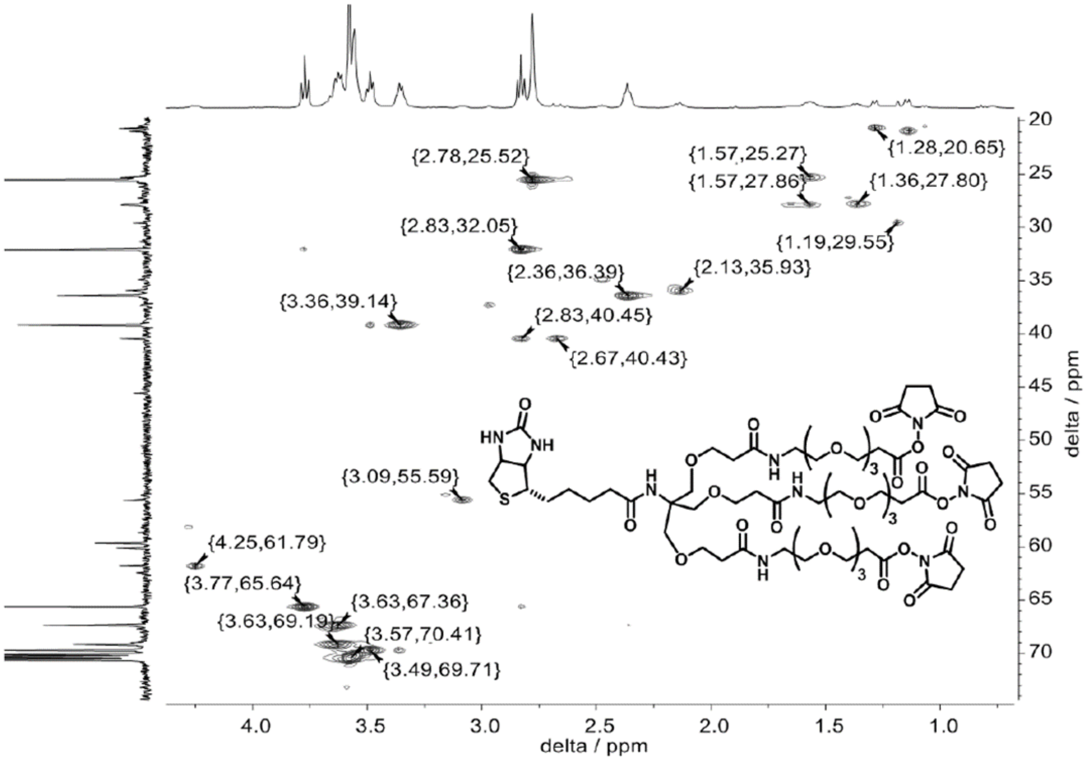

Figure S2. 2D-NMR experiment: heteronuclear single quantum correlation (HSQC) spectrum of Biotin$_{3xNHS}$X-Linker, 400 MHz, $CDCl_3$.

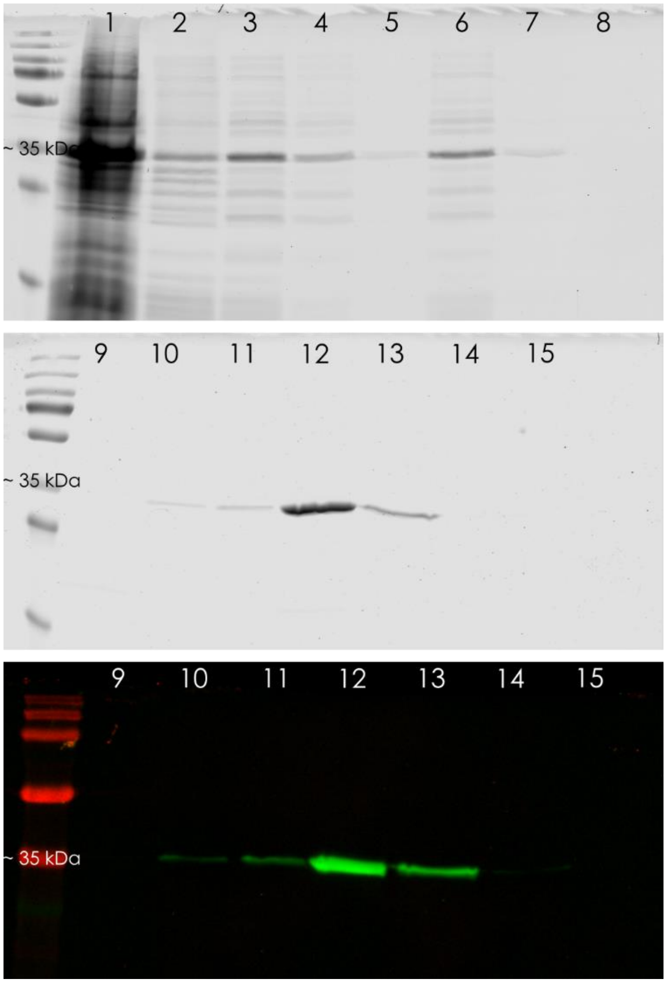

Figure S3. SDS-PAGE and Western Blot of AnxA2 WT purification steps. Lanes: 1) Supernatant lysis. 2) French press pellet. 3) DE flow through. 4) DE wash. 5) DE column material. 6) CM flow through. 7) CM buffer 1x. 8) CM buffer 2x. 9) CM 100 mM NaCl 1x. 10) CM 100 mM NaCl 2x. 11) CM 100 mM NaCl 3x. 12) CM 600 mM NaCl 1x. 13) CM 600 mM NaCl 2x. 14) CM 600 mM NaCl 3x. 15) CM column material. In the lower panel, immunoblotting with mouse monoclonal anti-AnxA2 antibodies directed against an N-terminal peptide (HH7 [22]). PageRuler Plus Prestained Protein Ladder (Thermo Scientific) was used as a marker.
